# DNA-Topology Simplification by Topoisomerases

**DOI:** 10.3390/molecules26113375

**Published:** 2021-06-03

**Authors:** Andreas Hanke, Riccardo Ziraldo, Stephen D. Levene

**Affiliations:** 1 Department of Physics and Astronomy, University of Texas Rio Grande Valley, 1 W University Blvd, Brownsville, TX 78520, USA; 2 Department of Bioengineering, University of Texas at Dallas, 800 W Campbell Road, Richardson, TX 75080, USA; Riccardo.Ziraldo@utdallas.edu; 3 Department of Biological Sciences, University of Texas at Dallas, 800 W Campbell Road, Richardson, TX 75080, USA; 4 Department of Physics, University of Texas at Dallas, 800 W Campbell Road, Richardson, TX 75080, USA

**Keywords:** DNA topology, type-II topoisomerases, site-specific recombination, master equations, non-equilibrium biophysics

## Abstract

The topological properties of DNA molecules, supercoiling, knotting, and catenation, are intimately connected with essential biological processes, such as gene expression, replication, recombination, and chromosome segregation. Non-trivial DNA topologies present challenges to the molecular machines that process and maintain genomic information, for example, by creating unwanted DNA entanglements. At the same time, topological distortion can facilitate DNA-sequence recognition through localized duplex unwinding and longer-range loop-mediated interactions between the DNA sequences. Topoisomerases are a special class of essential enzymes that homeostatically manage DNA topology through the passage of DNA strands. The activities of these enzymes are generally investigated using circular DNA as a model system, in which case it is possible to directly assay the formation and relaxation of DNA supercoils and the formation/resolution of knots and catenanes. Some topoisomerases use ATP as an energy cofactor, whereas others act in an ATP-independent manner. The free energy of ATP hydrolysis can be used to drive negative and positive supercoiling or to specifically relax DNA topologies to levels below those that are expected at thermodynamic equilibrium. The latter activity, which is known as topology simplification, is thus far exclusively associated with type-II topoisomerases and it can be understood through insight into the detailed non-equilibrium behavior of type-II enzymes. We use a non-equilibrium topological-network approach, which stands in contrast to the equilibrium models that are conventionally used in the DNA-topology field, to gain insights into the rates that govern individual transitions between topological states. We anticipate that our quantitative approach will stimulate experimental work and the theoretical/computational modeling of topoisomerases and similar enzyme systems.

## 1. Introduction

### 1.1. DNA Topology

The knot type, *K*, and the linking number, Lk, define the topological state of a single, covalently closed, double-stranded DNA circle. Furthermore, two or more DNA circles may form topologically interlocked molecular architectures of intertwined DNA circles, called catenanes (that are also referred to as links).

**Figure 1:**
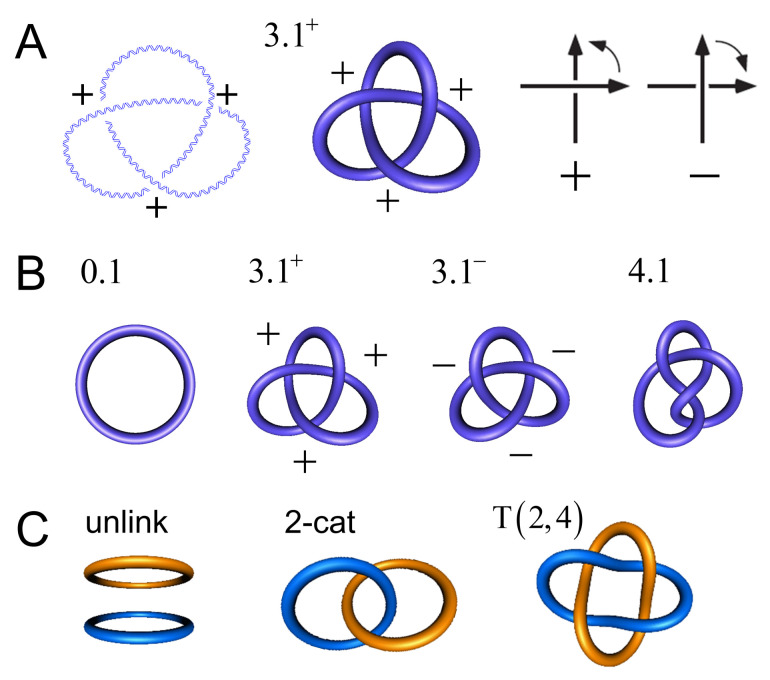
(**A**) Right-handed trefoil knot $3.1^{+}$ in double-helical DNA and its tube representation using knotplot (www.knotplot.com accessed on 16 May 2021). The sign convention for crossings in a planar projection is shown on the right. (**B**) Knots with minimal crossing numbers up to 4. From the left: The unknot 0.1, right-handed and left-handed forms of the chiral trefoil knot 3.1, and the amphichiral figure-eight knot 4.1. (**C**) Some low-order catenanes with two components. From left: The unlink, the 2-cat, and the torus catenane with two components and four crossings, T(2,4).

For circular DNA molecules (or a piece of string with joined ends), the knot type *K* is a topological invariant in the sense that it is maintained through all of the conformational changes that occur in the absence of breaking both strands of the DNA (or cutting the string). Most knots are chiral, which means that the knot and its mirror image form two topologically distinct enantiomorphic forms, whicha re referred to as right-handed and left-handed. The trefoil knot 3.1 is an example of a chiral knot, which occurs in the topologically distinct forms $3.1^{+}$ and $3.1^{-}$; one of these forms cannot be converted into the other without cutting the string forming the knot. Throughout this article, we use the Alexander–Briggs notation, in which the number 3 indicates the minimal crossing number (i.e., the minimal number of crossings in any knot diagram for the knot, equal to 3 for the trefoil knot), the number 1 is an index, and the superscript indicates whether the knot is right-handed (+) or left-handed (−) (Figure 1). An amphichiral knot is, by definition, topologically equivalent to its mirror image, i.e., the knot can be continuously deformed into its own mirror image. The lowest-order amphichiral knots are the unknot, 0.1, and the figure-eight knot, 4.1 (Figure 1B). The torus knots are a special subfamily of knots, which, by definition, can be wrapped on the surface of a torus in three-dimensional space without causing intersections. In the Alexander–Briggs notation, the torus knots are 0.1, 3.1, 5.1, 7.1, etc. All of the torus knots, except the unknot 0.1, are chiral.

**Figure 2:**
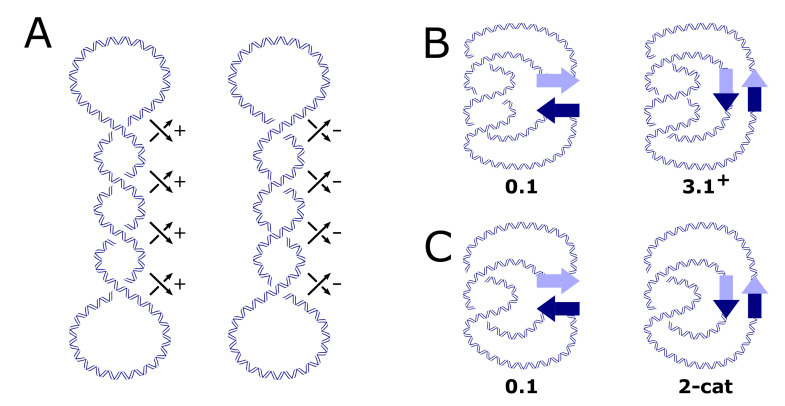
(**A**) Negatively and positively supercoiled DNA. (**B**) Supercoiling-assisted knotting of DNA circles by the action of a tyrosine recombinase on inversely repeated sites, converting an unknot into the right-handed trefoil knot, $3.1^{+}$. (**C**) Supercoiling-assisted catenation of DNA circles by the same reaction that i sshown in (**B**) taking place on directly repeated sites, converting an unknot into the two-crossing catenane or 2-cat. Note that the arrows in (**A**) indicate the direction of travel along the DNA defining the signs of the crossings (cf. Figure 1A), whereas the arrows in (**B**,**C**) indicate the DNA sequences of the recombination sites.

Two or more DNA circles may form catenanes of topologically interlocked DNA circles (Figure 1C). Similarly to torus knots, torus catenanes (or torus links) are a special subfamily of catenanes with the property that the strands of all circles forming the catenane can be wrapped on the surface of a torus in three-dimensional space without causing intersections. DNA torus catenanes are especially important in biology because they are formed between daughter DNAs during the replication of circular genomes; the catenation number, Ca, characterizes their topological state, which is the number of inter-duplex crossings analogous to the linking number, Lk, of the two complementary strands in the DNA double helix. Figure 2B,C show the DNA rearrangement that is mediated by a member of the tyrosine-recombinase superfamily. In this example, the protein binds to a circular DNA molecule, cuts the DNA at two specific sites, and subsequently exchanges and rejoins the cleaved ends, producing a knot or a catenane, depending on the orientation of recombination-target sites. With circular DNA, gel electrophoresis can analyze the generated distribution of topological products, which can resolve the distributions of knotted/catenated DNA circles according to the number of minimal crossings [1,2,3,4], and by electron microscopy [5,6,7].

For double-stranded DNA, the linking number Lk is given by the number of signed crossings of the two complementary strands in any planar projection of the double helix. For covalently closed DNA circles, Lk is, by definition, an integer and topologically invariant, i.e., is maintained through all conformational changes of the DNA that occur without breaking one or both DNA strands. By convention, the linking number Lk of a DNA molecule forming a right-handed double helix is positive. For a linear, mechanically relaxed DNA molecule, the linking number is given by a positive value $Lk_{0}=N/h_{0}$, where *N* is the number of base pairs in the DNA and $h_{0}$ is the number of base pairs per helical turn in mechanically relaxed DNA. For single circles of unknotted DNA, the degree of supercoiling is quantitatively defined in terms of the signed linking-number difference relative to relaxed DNA, $\Delta Lk=Lk-Lk_{0}$, rather than Lk itself. Supercoiling can also be expressed as a relative quantity through the superhelix density or a specific linking difference, $\sigma =\Delta Lk/Lk_{0}$. Negative supercoiling (ΔLk,σ<0) corresponds to undertwisting the DNA, i.e., a reduction in the linking number of the two complementary strands of the double helix relative to mechanically relaxed DNA. Conversely, positive supercoiling (ΔLk,σ>0) corresponds to overtwisting the DNA. The superhelical distortion is partitioned between an excess (+) or reduction (−) of DNA twist, Tw, the number of helical turns of the DNA about its center axis, and a coiling deformation of the DNA axis known as writhe Wr [8]. In any covalently closed DNA circle, the linking number, twist, and writhe are related by White’s Formula Lk=Tw+Wr, which may also be written as ΔLk=ΔTw+Wr, where ΔTw is the change in Tw relative to linear, mechanically relaxed DNA [8].

Since their discovery in the late 1960s, DNA knots and catenanes have been implicated in a number of cellular processes, including replication and recombination [8,9]. In vitro, DNA knots and catenanes are products of topological enzymology experiments on synthetic plasmid DNAs (Figure 2B,C), which provide insights into the binding and mechanisms of the enzyme being probed (see [10] and the references cited therein). Knots and links may also occur between more than two components of DNA. For example, a recent work demonstrated that knots and links between four-helices of DNA (that were made by self-assembled G-quadruplexes formed by GMP and Guanosine) can be attributed to the formation of highly hydrated gels [11].

As part of its role in regulating transcription, replication, and chromosomal segregation [12], DNA supercoiling has been proposed to play a role in the specificity of DNA-binding ligands, including major-groove binders, such as triplex-forming oligonucleotides [13,14]. These single-stranded oligonucleotides target specific DNA sequences, forming a triplex with the target duplex DNA [15]. Triplex-forming oligonucleotides have been developed as anticancer agents because of their target specificity and ability to suppress gene expression [16]. A recent study, combining atomic force microscopy (AFM) and atomistic molecular dynamics (MD) simulations, found that negative superhelical stress induces local variations in the canonical B-form DNA structure by introducing local kinks and defects, which significantly affect the binding of triplex-forming oligonucleotides to DNA minicircles [17]. Thus, DNA supercoiling directly affects the molecular recognition of DNA by DNA-binding ligands.

### 1.2. Roles of DNA Supercoiling in the Topological Organization in Genomes

**Figure 3:**
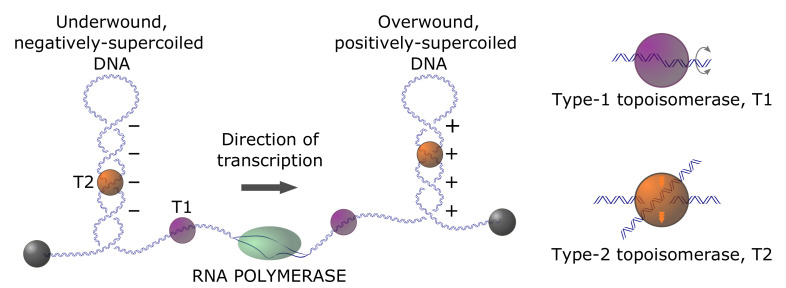
The twin-supercoiling-domain model proposed by Liu and Wang (1987) [19]. As the coupled transcription complex moves from left to right, the DNA template ahead becomes overwound (positively supercoiled plectonemes), while the DNA behind becomes underwound (negatively supercoiled plectonemes). Regions of naked DNA are preferentially unwound by type-I topoisomerases whereas DNA within supercoiled plectonomes is preferentially unwound by type-II topoisomerases (adapted from [24]).

In prokaryotes, there is compelling evidence for the homeostatic regulation of in-vivo supercoiling, such that the average, steady-state superhelix density, σ, is around one negative twist per 400 base pairs (σ≈−0.03) [5]. This is the level of unconstrained (free) supercoiling; an approximately equal level of supercoiling is accounted for by wrapping genomic DNA around specifically and non-specifically bound proteins. Unconstrained supercoiling is, in principle, free to diffuse within topologically closed domains, corresponding to regions of the genome that are constrained through physical clamping via interactions between the genomic DNA and DNA-binding proteins or other subcellular structures, or through limited rotational motion. Supercoiling is intimately connected with genome dynamics via long-range three-dimensional (3D) conformational changes of genomic DNA, (un)twisting by protein complexes that track along the DNA duplex, and localized changes in a helical structure, such as cruciform formation or B-to-Z transitions. Transcriptional activity generates positive and negative waves of supercoiling in topologically closed domains ahead of and behind the transcription apparatus, respectively, according to the twin-domain model of Liu and Wang (Figure 3) [18,19]. Thus, supercoiling that is associated with transcriptional activity potentiates local and global changes in genome architecture [5,20,21,22]. Therefore, the dynamic behavior of the genome in this context depends on both the regulation of supercoiling (via, e.g., topoisomerase-dependent relaxation activity) and time-varying topological constraints [23].

Although it is generally accepted that the organization of eukaryotic genomes is hierarchical, our understanding of higher-order DNA organization (beyond the nucleosome level) is progressively more limited with an increasing length scale. The amount of steady-state unconstrained supercoiling in eukaryotic genomes has not been definitively established and it remains controversial [25,26,27]. This is, in part, due to the technical challenge of measuring supercoiling in a dynamic chromatin environment. Much of what we know regarding spatial organization in eukaryotic genomes comes from chromatin-conformation-capture (3C) experiments and elaborations thereof (4-C, 5-C, Hi-C, etc.) [28,29,30]. The pictures that emerge from multi-C experiments are presently limited in resolution (25-kbp to 1-Mbp), and they do not provide insight into the local details of DNA supercoiling. The identification of “topologically associated domains” (TADs) in mammalian cells and “chromosomal interacting domains” (CIDs) in other organisms has been a major breakthrough of such methods [31]. These domains are defined in terms of chromatin loops involving long curvilinear distances along the genome and they are likely mediated by CCCTC-binding factor (CTCF), cohesin, condensin, mediator, and possibly other proteins [31]. Th ephysical proximity of sequences at the loop termini is neither a necessary nor sufficient condition to guarantee the topological isolation of a domain. Genomic sequences can be physically proximal simply because of constraints related to nuclear packing, for example [26]. Single-cell Hi-C data underscore the dynamic nature of genome architecture in haploid mouse cells [32]. The analysis of single cells, as opposed to populations, reveals substantial cell-to-cell variations in putative inter- and intra-chromosomal contacts. More quantitative approaches for analyzing multi-C data are needed, but they have been slow to materialize [33]. Computed 3D structures of individual chromosomes in the nuclear milieu have been derived from extensive computational modeling of Hi-C contact maps using polymer models of the chromatin fiber. The resulting limited-resolution models (polymer-segment size ∼100 kbp) have been interpreted as consistent with the knotting of intra-chromosomal domains and/or topological entanglements between chromosomes [32]. Experimental techniques have not yet been brought to bear on the question of whether these topological linkages truly exist.

### 1.3. Structure and Mechanism of DNA Topoisomerases

**Figure 4:**
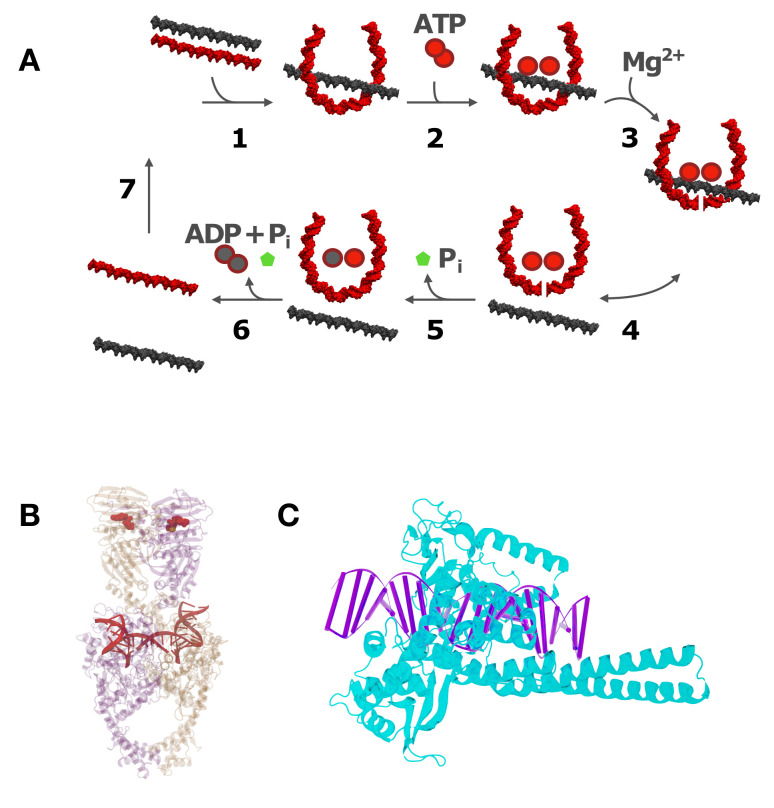
(**A**) The steps of duplex-DNA passage in type-II topoisomerases catalyzed by hydrolysis of two ATP molecules and binding of two Mg${}^{2+}$ ions. The gate (G) segment of the DNA is shown in red and the transfer (T) segment in black. (**B**) Structural model of yeast type-II topoisomerase bound to G-segment DNA and adenylyl-imidodiphosphate (AMP-PNP), a non-hydrolysable analogue of ATP. In this system, the DNA is bent by 160 ${}^{\circ }$ [40]. (**C**) Co-crystal structure of human type-I topoisomerase (PDB 1A36) with DNA. Both of the crystal structures were obtained from RCSB (www.rcsb.org, 16 May 2021). The images were adapted using Jmol (www.jmol.org, 16 May 2021) and Blender (www.blender.org, 16 May 2021).

There are two broad, but distinct, classes of DNA topoisomerases: type-I enzymes, which alter Lk (and, therefore, ΔLk) in steps of one [34,35], and type-II enzymes, which alter Lk in steps of two [36,37]. Type-I enzymes remove torsional stress by creating a DNA nick, i.e., cutting one of the two DNA strands, thus allowing the free ends of the nicked strand to swivel about the DNA center axis (Figure 3). In this reaction, ΔLk exclusively changes by ±1 and leaves the knot type *K* intact (however, it is known that *E. coli* topoisomerase I can perform duplex strand passage at a nick and, therefore, can alter the knot type *K* [38]). Type-II enzymes are divided into two subfamilies: type IIA (topoisomerases II and IV) and type-IIB (topoisomerase VI). They can change both *K* and ΔLk by passing one duplex-DNA segment through a duplex-DNA break (Figure 3 and Figure 4). There is a change in ΔLk of ±2 for every reaction cycle when type-II enzymes act intramolecularly on circular DNA; for intermolecular reactions, the catenation number, Ca, changes by ±2 per cycle. At least one type-II topoisomerase homolog is expressed in every known organism [39].

All of the known type-II enzymes use ATP as an energy cofactor [41], although the detailed mechanistic role of this cofactor is variable and remains controversial in the majority of cases. A special class of type-IIA enzymes, bacterial gyrases, generate negative supercoils at the expense of ATP hydrolysis [42,43,44,45]. Type-II topoisomerases carry out strand passage via a gated mechanism, wherein a double-strand break (DSB) is created by active-site tyrosine residues at a “gate” or “G” DNA segment bound to the TOPRIM (topoisomerase-primase) domain of the enzyme (Figure 4A) [46,47]. A DNA segment (the “T” segment) is captured by ATP-dependent protein interactions in the N-terminal domain of the protein (Figure 4A, step 2) in the first stage of a multiple-clamp mechanism [48]. The T segment is then passed through the break, followed by the resealing of the G segment. Type-II enzymes can vary according to whether an additional gated domain (the “C gate”) receives the passed T segment [8,39], and in their preferences for relaxing DNA crossings of different chiralities [49,50,51]. However, only bacterial DNA gyrase has the ability to actively introduce negative supercoils into DNA at the expense of ATP hydrolysis [52].

The precise role of ATP hydrolysis in type-II reactions is controversial. One view is that the coupling of enzyme action to ATP degradation is necessary for driving levels of DNA supercoiling, linking, and knotting below those at thermodynamic equilibrium, a phenomenon that is referred to as "topology simplification” (see Section 1.4, below) [9,53]. A different, but compelling, argument can be made that ATP hydrolysis is tightly coupled to the gating of DNA segments through transient double-stranded breaks in order to ensure that the break is resealed before the transported segment is released (Figure 4A, step 5). There is strong selective pressure for such a mechanism, since the presence of double-stranded breaks is highly deleterious, indeed fatal, to the cell [54,55].

Type-I topoisomerases (Figure 4C) are ATP-independent enzymes that relax both positive (+) and negative (−) supercoils, except for reverse gyrases, which are found in archaea and some bacteria. The torsional relaxation of DNA is thermodynamically favorable and, therefore, can occur spontaneously without an energy cofactor (Figure 3). Reverse gyrases are type-I enzymes that use the free energy of ATP hydrolysis to introduce positive supercoils into DNA [56]. These topoisomerases are single polypeptides that contain a fusion of a type-IA topoisomerase domain and a helicase domain [57,58,59]. The introduction of (+) supercoils generates an overwinding of the DNA duplex, which dramatically increases the free energy of strand separation. Resistance to strand separation is a selective advantage for organisms that thrive at high ambient temperatures, such as undersea thermal vents [60].

### 1.4. Biological Roles of Type-II Topoisomerases

During transcription, the rotation of the DNA duplex relative to the RNA polymerase produces positive DNA supercoiling in front of the DNA transcription machinery and negative supercoiling behind it (Figure 3) [18,19,61]. Eukaryotic RNA polymerases transcribe DNA at rates of ∼100 bp/s, which implies that, locally, DNA becomes transiently overtwisted and undertwisted, respectively, by approximately 10 turns/s [62]. The major role of type-II topoisomerases in transcription was thought to be the relaxation of excess supercoiling ahead of and behind the transcription machinery [63,64]. Type-II enzymes have a similar role in regulating supercoiling that is generated during DNA replication [65,66]. However, it was recently found that a combination of positive DNA supercoiling in front of the transcription complex and chromatin compaction dramatically increases the level of type-II enzyme-mediated knotting of DNA [67]. In general, supercoiling increases the effective local concentration of DNA segments in the vicinity of one another [68], which correspondingly increases the probability of local type-II enzyme-mediated segment-passage events. Indeed, type-II topoisomerases produce a large variety of complex DNA knots in vitro when DNA is compacted by supercoiling or agents that promote DNA condensation [5,69,70]. This picture is not significantly altered in chromatin structures under conditions, such that nucleosome arrays are compacted by supercoiling or other mechanisms. Ultimately eliminating knotted DNA products, once formed, is a critical biological function of type-II enzymes, since knotting blocks transcription and replication, which results in cell death [71,72,73,74]. Thus, although relaxation of supercoiling can, in principle, be carried out by either type-I or type-II enzymes, only type-II enzymes can provide the duplex-segment passage that is required for unknotting of DNA domains or the decatenation of circular genomes, which is an essential step in the cell division required for proper chromosome segregation [75,76].

Knotting and catenation are byproducts of other extrinsic processes that generate non-trivial DNA topologies. DNA site-specific recombination is an example of such a process, which is reasonably well understood in the case of the tyrosine and serine site-specific recombinases (Figure 2B,C) [4,77,78]. Site-specific recombination reactions can create torus knots with three and more irreducible crossings [79,80,81]. Similarly, transposases [82,83,84] and the V(D)J recombination system [85,86,87] can also generate non-trivial DNA topologies. Furthermore, in vitro, it was shown that type-IIA topoisomerase can produce knotted DNA in cases where DNA segments far apart along the DNA are brought in close physical contact by DNA looping, supercoiling, or interactions with DNA-condensing agents [19,69,70].

Type-II enzyme action is no less relevant for organisms with linear genomes. This is due to the organization of large linear chromosomes as topologically isolated domains through intra- and inter-chromosomal protein-protein and protein-DNA interactions [88,89,90,91]. Such isolated domains are subject to topological constraints that are similar to those of circular chromosomes. The unknotting of these domains by type-II topoisomerases may also be biologically important [92]. A recent computational study found that structural-maintenance-of-chromosomes (SMC) proteins, such as cohesins and condensins [93,94,95,96], may cooperate with type-II enzymes to resolve topological DNA entanglements [97]. According to this mechanism, SMC-driven loop extrusion induces a spatial localization of essential crossings of the topological entanglement, which, in turn, facilitates the simplification of knots and links by type-II enzymes even in crowded and confined conditions [97].

All of the type-II topoisomerases are strictly dependent on ATP hydrolysis to carry out the net relaxation of supercoils and resolution of knot or catenane topologies (except bacterial gyrases, which generate negative supercoils at the expense of ATP hydrolysis [42,43,44,45]). Rybenkov et al. showed that this reduction in topological complexity can exceed, by a factor of up to 80, the level expected to occur if the system was simply allowed to attain thermodynamic equilibrium [53]. The excess reduction in the variance of topological parameters is termed “topology simplification” [9,53], and it is potentially biologically significant because the linkage between daughter chromosomes in a dividing cell must be reduced exactly to zero in order for proper chromosome segregation to occur. This means that, for circular genomes, *Ca* must be exactly equal to zero. An enzyme cannot drive a system into such a non-equilibrium state without thermodynamically coupling the enzymatic activity to another process that provides a free-energy source. Moreover, it is remarkable that an enzyme that is quite small relative to its DNA substrate can selectively carry out DNA-segment passages in a way that simplifies global DNA topology. The latter is a property of the entire molecule and not local DNA-enzyme interactions [53,98,99,100,101,102,103].

It is useful to draw a functional distinction between enzymes that carry out topoisomerase activity in the absence of an energy cofactor, and those that depend on an energy cofactor, such as ATP. The former category consists of all type-I enzymes, except for reverse gyrase; the latter includes type-I reverse gyrase, gyrase, and all other type-II enzymes. Cofactor-independent enzyme pathways must follow the free-energy gradient in traversing the topological landscape between the initial and final topological states. In contrast, cofactor-dependent topoisomerases can follow alternative pathways that depend, in detail, on how the free energy of cofactor hydrolysis is utilized. In Section 2 and Section 3, we focus on the non-equilibrium aspects of type-II topoisomerase mechanism and emphasize the general principles that would apply to any cofactor-dependent enzyme system that modulates DNA topology.

Because of their essential biological roles, the drug targeting of topoisomerase mechanisms has been a highly successful strategy for developing antimicrobial drugs and cancer chemotherapies. There are two classes of drugs that disrupt type-II topoisomerase activity: topo-II poisons and topo-II inhibitors [104]. Poisons stabilize the G-segment cleavage complex (the product of step 3 shown in Figure 4A). This leads to irreversible formation of a covalent topoisomerase-DNA intermediate, the topo-II cleavage complex (Top2cc). Inhibitors prevent the formation of Top2ccs by interfering, with DNA binding or blocking, the double-strand cleavage reaction. Etoposide and the anthracycline doxorubicin are among the most commonly used anti-neoplastic drugs. These topo-II poisons act on cancer cells through enzyme-mediated DNA damage; however, the mechanistic details remain controversial [105]. The destruction of cancer cells by these drugs is accompanied by extensive collateral damage to healthy cells, and the repair of drug-mediated double-stranded DNA breaks present a long-term risk of disease recurrence for cancer survivors who have undergone chemotherapy [106]. The inhibition of topoisomerase activity without the formation of double-stranded breaks can also have deleterious effects. Reduced levels of topoisomerase activity (topoisomerase I and topoisomerase IIB) have been suggested to play a role in autism spectrum disorder through the dysfunctional expression of long genes [107]. This can be rationalized in terms of transcriptional inhibition due to insufficient rates of superhelix relaxation. The factors depressing topoisomerase activity in this case remain to be identified.

### 1.5. DNA-Topology Simplification by Type-II Topoisomerases

Several models have been proposed to explain the resolution of DNA knots and catenanes below levels at thermodynamic equilibrium while using the energy that was provided by ATP hydrolysis [53,98,99,100,101,102,108,109,110,111,112,113,114]. Because type-II topoisomerases are much smaller than the DNA molecules they act on (typically kbp in size and larger), the enzymes cannot directly determine the DNA knot type since the latter is a topological property of the entire DNA molecule. Therefore, the proposed models generally use the idea that the enzyme probes *local* statistical properties of the DNA that depend on the *global*, topological state of the DNA. A successful example for such a model is the "hairpin-like” G segment, in which the type-II enzyme creates a sharp bend in the G segment, which results in the unidirectional passage of the T segment from the inside to the outside of the hairpin-like G segment (Figure 4) [98,99]. Indeed, the formation of a sharply bent enzyme-bound G segment is supported by topoisomerase II in yeast DNA (Figure 4B) [115] as well as AFM and FRET measurements [116]. Thus, type-II enzymes act like Maxwell’s demon, which only allows those strand passages that change DNA topology in a desired direction, under the consumption of energy that is provided by ATP hydrolysis [98,99,101,102,103].

In order to study the non-equilibrium dynamics of transitions between topological states in circular DNA molecules by type-II enzymes (and by other enzymes that change DNA topology, such as site-specific recombinases, cf. Figure 2B), we introduce a network of DNA topological states (K,ΔLk), where the transitions between these states that are catalyzed by the enzyme are described by a chemical master equation (Section 2.1) [103]. This is a multiscale approach that uses DNA-topological states as the variable on the macroscopic level and transition rates that depend on molecular details, such as geometric features of type-II enzyme action on the microscopic level. For comparison, we also consider torsionally unconstrained (nicked) DNA circles, for which the topological states are defined by the knot type *K* alone. Such a system can be realized by the combined action of type-II and type-I enzymes. Previous studies showed the existence of unknotting/unlinking pathways that are generated by type-II enzyme action that progressively reduces the topological complexity of knotted/catenated molecules in a stepwise manner [49,79]. The main goal of our study is to identify significant pathways along which topology simplification by type-II enzymes occurs in the network (K,ΔLk). To this end, we generated a large set of equilibrium ensembles of knotted and supercoiled 6-kbp DNAs by Monte Carlo simulations to find microscopic transition rates, which are then used in a chemical master equation to find non-equilibrium steady-state probabilities and steady-state currents for topological states (K,ΔLk) (Section 2 and Section 3). An important feature of our model is that it can incorporate extrinsic biological processes that actively generate knots and/or supercoils in DNA (Section 1.4).

## 2. Methods

### 2.1. Chemical Master Equation for Transitions between DNA Knot Types

We consider an ensemble of uncatenated DNA circles of fixed length in dilute solution at the concentration $c_{0}$. For large DNA molecules that are several kbp in size, the average dimensions of DNA conformations are much larger than those of enzymes that act on the DNA, such as DNA topoisomerases and recombinases. For simplicity, we first consider torsionally unconstrained DNA, for which the linking number difference ΔLk may vary freely. Torsionally unconstrained DNA can result from irreversible single-strand scission (nicks) due to enzymatic or physico-chemical processes, or transiently from the action of type-I topoisomerases (Section 1.3). In this case, the topological state of a circular DNA molecule is entirely specified by the knot type *K*, which is preserved in the absence of reactions that cut both duplex-DNA strands. As a result, the initial distribution of knot types in the solution is preserved. That is, the particular concentrations c(K) of DNA molecules having knot type *K* remain fixed at the initial values $c_{0}\left(K\right)$.

However, in the presence of enzymatic reactions that change the knot type *K*, the concentrations c(K) are no longer fixed, but they depend on time *t* in general, i.e., they become time-dependent functions c(K,t). Examples of enzymes that can change the DNA knot type are type-II topoisomerases and site-specific recombinases acting on inversely repeated sites (Figure 2B; for simplicity, we only consider intramolecular reactions, as shown in Figure 2B. We do not consider the formation of catenanes, which could occur, in principle, at high enough DNA concentrations for independent molecules to interact). In this scenario, the change in c(K,t) for a particular knot type *K* is determined by a balance of reactions $K^{\prime }\to K$ and $K\to K^{\prime }$ that are catalyzed by the enzyme, where $K^{\prime }$ denotes any knot type that is different from *K*:

(1) $$\frac{d}{dt}c\left(K,t\right)=\sum_{K^{\prime }\neq K}\left[c\left(K^{\prime },t\right)k\left(K^{\prime },K\right)-c\left(K,t\right)k\left(K,K^{\prime }\right)\right]\equiv \sum_{K^{\prime }}W\left(K,K^{\prime }\right)c\left(K^{\prime },t\right)\;.$$

An equation of this type is referred to as chemical master equation. The sum in the expression in the middle of Equation (1) is over all knot types $K^{\prime }$ different from *K* and $k(K,K^{\prime })$ is the rate constant for an enzymatic reaction that converts a DNA molecule having knot type *K* to a DNA molecule having knot type $K^{\prime }$, which corresponds to a reaction $K\to K^{\prime }$. More precisely, $k(K,K^{\prime })$ is the fraction of DNA molecules having knot type *K* that are converted to knot type $K^{\prime }$ per unit time; equivalently, $k(K,K^{\prime })$ is the probability that an individual DNA molecule having knot type *K* is converted to knot type $K^{\prime }$ per unit time. The sum on the right-hand side of Equation (1) is over all knot types $K^{\prime }$ (including *K*) and the matrix $W(K,K^{\prime })$ is given in terms of the transition rates $k(K,K^{\prime })$. Note that the transition rates $k(K,K^{\prime })$ are assumed to be time-independent for all knot types $K,K^{\prime }$. This implies the following assumptions:

**Assumption** **1.** *The activity and concentration of the enzymes catalyzing the reaction $K\to K^{\prime }$ are constant. Likewise, the total concentration $c_{0}$ of DNA molecules participating in the enzymatic reactions is constant.*

**Assumption** **2.** *Equation (1) implies a separation of time scales: the conversion of knot types $K\to K^{\prime }$ by the enzyme is a slow process compared to conformational rearrangements of the DNA molecules due to thermal fluctuations [117]. Thus, the time evolution of c(K,t) shown in Equation (1) describes the slow process of conversions of DNA knot types *K* by the enzyme, whereas the fast process of conformational rearrangements of the DNA due to thermal fluctuations determines the rate constants $k(K,K^{\prime })$ according to Assumption 3, below.*

**Assumption** **3.** *The assembly of the enzyme-DNA complex resulting in a reaction $K\to K^{\prime }$ is not a diffusion-limited process. This implies that the rates $k(K,K^{\prime })$ are proportional to the probability that a DNA having knot type *K* takes on a conformation that enables the enzymatic reaction in the DNA conformational space. This probability will be referred to as the juxtaposition probability below. According to Assumption 2, the enzymatic reaction is a quasi-static process, in the thermodynamic sense, when compared to DNA conformational rearrangements. This implies that the juxtaposition probabilities determining the transition rates $k(K,K^{\prime })$ for reactions $K\to K^{\prime }$ can be determined from an equilibrium ensemble of DNA conformations with a fixed knot type *K* (see Equation (5) below).*

**Figure 5:**
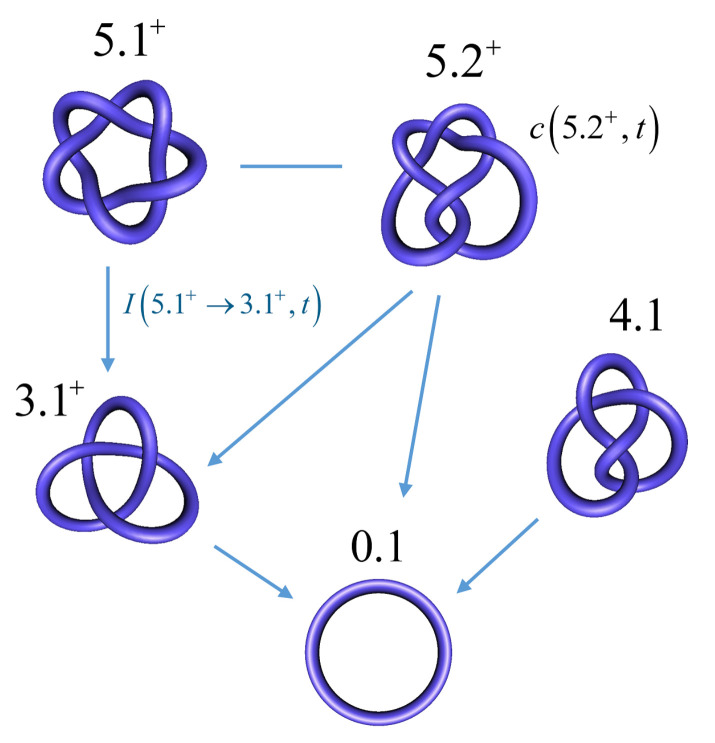
Transitions between topological states in DNA corresponding to fixed knot types *K* catalyzed by type-II enzyme. Time-dependent specific concentrations of each knotted species c(K,t) are described by the chemical master Equation (1). The blue lines represent possible transitions by single passages of the DNA double helix (one-passage connectivity). The transitions are biased towards the unknot as indicated by the arrows (topology simplification).

It is important to note that Equation (1) describes a *time-dependent, non-equilibrium dynamic process* of conversions of DNA knot types *K*, although the rate constants $k(K,K^{\prime })$ are time-independent due to a separation of time scales (Assumption 2). The description of non-equilibrium reaction dynamics in terms of chemical master equations using time-independent rate constants is a well-known approach in chemical and enzyme kinetics [118,119,120]. Figure 5 illustrates the transitions between some simple (low-complexity) knot types *K* in circular DNA that is catalyzed by type-II enzymes. Each knot type *K* represents a topological state with an associated equilibrium ensemble of DNA conformations having this knot type *K* (due to Assumption 2). Type-II enzymes can induce transitions between these topological states, which results in time-dependent concentrations c(K,t) and transition currents of DNA from one topological state (knot type *K*) to another (cf. Equation (4), below). The chemical master Equation describes the non-equilibrium dynamics of these transitions (1).

An important special case is one in which the particular concentrations c(K,t) are stationary. This case is realized in the absence of reactions that change the DNA knot type, but may also result from a dynamic equilibrium between transitions $K^{\prime }\to K$ and $K\to K^{\prime }$. In that instance the right-hand side of Equation (1) vanishes, although the transition rates $k(K,K^{\prime })$ are finite, i.e., reactions $K\to K^{\prime }$ do occur. This implies that $\frac{d}{dt}c\left(K,t\right)=0$, so that the partial concentrations c(K) are constant (independent of time). This situation is realized in two general cases (cf. Section 1.4):

Case 1: Thermal Equilibrium. The DNA-knot changing reactions $K\to K^{\prime }$ occur in the absence of external energy sources, i.e., in the absence of ATP hydrolysis and other reactions that contribute external energy to the system. In this case, for thermodynamic reasons, the time-dependent probabilities c(K,t) shown in Equation (1) approach, after some time, the thermal equilibrium probabilities that are determined by the Boltzmann factor,

(2) $$c\left(K,t\right)\to c_{eq}\left(K\right)∼\exp\left(-\frac{F(K)}{k_{B}T}\right)\;,$$

where F(K) is the free energy of a DNA circle forming knot type *K*, *T* is the temperature in Kelvin, and $k_{B}$ is the Boltzmann constant. A sufficient (but not necessary) condition for the right-hand side of Equation (1) to vanish for constant c(K) is the *condition of detailed balance*

(3) $$c\left(K^{\prime }\right)k\left(K^{\prime },K\right)=c\left(K\right)k\left(K,K^{\prime }\right)\;\mathrm{for}\;\mathrm{all}\;K,K^{\prime }.$$

Equation (3) implies that, for each pair of knot types *K*, $K^{\prime }$, the number of DNA molecules having knot type $K^{\prime }$ that are converted to *K* (reaction $K^{\prime }\to K$) per unit time and unit volume is the same as for the reverse reaction $K\to K^{\prime }$. In general, the condition of detailed balance holds for the equilibrium distribution $c_{eq}\left(K\right)$ shown in Equation (2).

Case 2: Non-Equilibrium Steady States (NESS). The DNA-knot changing reactions $K\to K^{\prime }$ occur in the presence of external energy provided, e.g., by the hydrolysis of ATP. In this case, the time-dependent partial concentrations c(K,t) in Equation (1) may approach, after some time, constant, steady-state concentrations $c^{*}\left(K\right)$, which are not necessarily equal to the equilibrium concentrations $c_{eq}\left(K\right)$ (the star symbol for $c^{*}\left(K\right)$ is used to distinguish steady-state concentrations from equilibrium concentrations $c_{eq}\left(K\right)$). This is the case for DNA strand passages by type-II topoisomerases that consume energy by hydrolysis of ATP and produce steady-state concentrations $c^{*}\left(K\right)$ different from $c_{eq}\left(K\right)$, namely being biased towards the unknot (topology simplification) [53]. It is notable that. for steady-state concentrations $c^{*}\left(K\right)$, the right-hand side of Equation (1) still vanishes (as for the case at thermodynamic equilibrium); however, the detailed balance condition in Equation (3) is violated in general. This implies the possibility of nonvanishing steady-state “currents”

(4) $$I^{*}\left(K\to K^{\prime }\right)\equiv c^{*}\left(K\right)k\left(K,K^{\prime }\right)-c^{*}\left(K^{\prime }\right)k\left(K^{\prime },K\right)\;,$$

that correspond to the net number of DNA molecules with knot type *K* that are converted to knot type $K^{\prime }$ per unit time and unit volume (cf. Figure 5, which shows such a current for the general, non-stationary case). The net current may be directed from *K* to $K^{\prime }$ (+) or from $K^{\prime }$ to *K* (−), depending on the sign of $I^{*}\left(K\to K^{\prime }\right)$. These currents may form directed cycles, e.g., $K\to K^{\prime }\to K^{\prime \prime }\to K$, which are characteristic of a system of non-equilibrium steady states. Note that the steady-state concentrations $c^{*}\left(K\right)$, although being constant, belong to an inherently *non-equilibrium* system, because they are dynamically maintained by the ongoing provision of external free energy, e.g., by the hydrolysis of ATP that drives the system away from thermal equilibrium. The study of such non-equilibrium steady states (NESS) is a very active field in non-equilibrium thermodynamics, with numerous recent applications to biological systems [94,121,122,123,124,125,126,127,128,129].

To be specific, in what follows, we consider DNA strand passages that are catalyzed by type-II topoisomerase enzymes that may generate transitions $K\to K^{\prime }$ of DNA knot types (Section 1.3). The associated transition rates $k(K,K^{\prime })$ that are shown in Equation (1) are assumed to be of the form [98,99,101,103]

(5) $$k\left(K,K^{\prime }\right)=k_{0}j\left(K\right)Q\left(K^{\prime }|K\right)\;,$$

where j(K) is the juxtaposition frequency of the DNA-bound enzyme for DNA having knot type *K*, which corresponds to the fraction of DNA conformations in which a potential T segment is properly juxtaposed with the G segment (Section 1.3). $Q(K^{\prime }|K)$ is the conditional probability that a strand passage from a juxtaposed DNA conformation having knot type *K* results in knot type $K^{\prime }$. The constant $k_{0}$ depends on enzyme activity and concentration, but it is independent of the knot types, $K,K^{\prime }$ of reactant and product DNA, respectively [98,99,101,103].

### 2.2. Computational Procedure and Model of Type-II Enzymes

**Figure 6:**
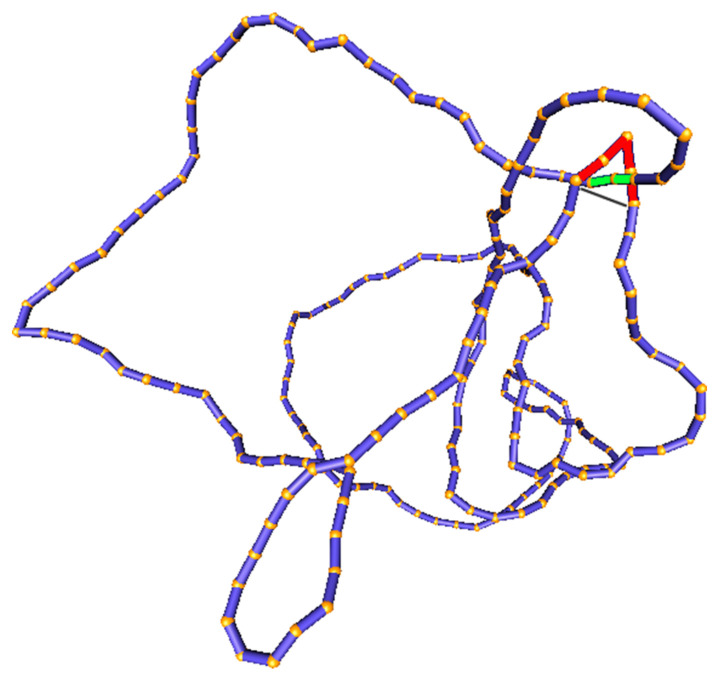
The simulation snapshot of the discrete wormlike chain model for duplex DNA forming a left-handed trefoil knot $3.1^{-}$. The hairpin-like G segment used to model the type-II enzyme is shown in red [134]. In the depicted conformation, a T segment (green) is properly juxtaposed with the G segment (red) to initiate the strand passage. The virtual deformation of the chain used to determine the product knot type $K^{\prime }$ after strand passage is indicated by the gray line.

As pointed out above (as in Section 2.1, Assumption 2), the transition rates $k(K,K^{\prime })$ in Equation (5) can be determined from an equilibrium ensemble of DNA conformations with fixed knot type *K*. Such an equilibrium ensemble can be generated by a Monte Carlo computer simulation of a semiflexible, discrete wormlike chain model for duplex DNA, which consists of a chain of straight, impenetrable cylindrical segments of diameter *d* with a specified bending rigidity between successive segments [68,130,131,132] (Figure 6). This model accurately describes the transition probabilities between different knot types in plasmid-sized DNA circles, which are typically several kbp in size, since the knot type is a topological property of the entire DNA molecule and is independent of the microscopic details of the DNA double helix. The simulation generates a sequence of DNA conformations by forming trial conformations at each Monte Carlo step, which are accepted or rejected according to the Metropolis criterion [68,130,131,132]. The bending rigidity between successive segments is adjusted, so as to reproduce the persistence length, *P*, of the DNA under given conditions, e.g., P∼ 50 nm (150 bp) for B-form DNA under physiological conditions. The effective segment diameter *d* is used to model the excluded-volume and electrostatic interactions between DNA segments. Because counterions in the solution screen the negatively charged DNA segments, thereby reducing the electrostatic repulsion between them, the value of *d* strongly depends on the ionic conditions of the solution. Thus, increasing the salt concentration reduces the effective segment diameter *d*. The value of *d* for given salt concentration is obtained from experimental and theoretical studies [53,117]; e.g., d= 5 nm for an ionic strength of 150 mM [117]. A given knot type *K* is preserved during the simulation by calculating the Alexander polynomial and HOMFLY polynomial for every trial conformation, and rejecting any trial conformation having a different knot type than *K* [133].

In our simulations [103], DNA-bound type-II enzymes with hairpin G segments were modeled by selecting four contiguous chain segments that form two sides of an equilateral triangle, corresponding to a 120${}^{\circ }$ bend. A putative T segment was considered to be juxtaposed with the G segment if it passed through the triangle in such a way that none of the chain segments overlapped (Figure 6). The juxtaposition probability j(K) shown in Equation (5) was calculated as the fraction of DNA conformations with fixed knot type *K* that fulfill the juxtaposition condition that is described above. The conditional transition probability $Q(K^{\prime }|K)$ presented in Equation (5) was obtained by determining the knot type $K^{\prime }$ that would result from the juxtaposed DNA conformation (having knot type *K*) by the passage of the T segment through the G segment. To this end, we considered local, virtual deformations of the chain that was obtained by replacing the red segments that correspond to the G segment by the gray segments that are shown in Figure 6, and determined the knot type $K^{\prime }$ for the virtually deformed chain using the Alexander and HOMFLY polynomials. $Q(K^{\prime }|K)$ was then calculated as the fraction of product knot types $K^{\prime }$ found for the set of juxtaposed conformations obtained in the simulation of a DNA molecule having knot type *K*.

## 3. Results

### 3.1. Steady-State Distribution of Knots in Torsionally Unconstrained DNA

We first consider torsionally unconstrained (due to nicks or type-I enzyme action) 6-kbp circular DNA at concentration $c_{0}$ in the presence of type-II enzymes and ATP in order to illustrate unknotting of circular DNA by type-II enzymes. Without other components, the topoisomerase action will keep the steady-state probability of knotted DNA below thermal equilibrium, implying a very low occurrence of any knot type other than the unknot, as reported previously [53,98,99,101,103]. In order to show this, it is convenient to write the partial concentrations c(K,t) in Equation (1) as

(6) $$c\left(K,t\right)=c_{0}\frac{c(K,t)}{c_{0}}\equiv c_{0}P\left(K,t\right)\;,$$

where $P\left(K,t\right)=c\left(K,t\right)/c_{0}$ is the probability that an individual DNA molecule in the solution has knot type *K* at time *t*. These probabilities are normalized as $\sum_{K}P\left(K,t\right)=1$ for any time *t* (hence, can be expressed in percent) and they obey the master equation

(7) $$\frac{d}{dt}P\left(K,t\right)=\sum_{K^{\prime }}W\left(K,K^{\prime }\right)P\left(K^{\prime },t\right)\;,$$

with the same transition matrix $W(K,K^{\prime })$ as in Equation (1). Stationary NESS probabilities $P^{*}\left(K\right)$ were calculated by using the condition $\sum_{K^{\prime }}W\left(K,K^{\prime }\right)P^{*}\left(K^{\prime }\right)=0$ for all knot types *K* that are accessible to the system, which implies that the vector $\left[P^{*}\left(K^{\prime }\right);\mathrm{knot}\;\mathrm{types}\;K^{\prime }\right]$ is an eigenvector of the transition matrix $W(K,K^{\prime })$ with eigenvalue 0 [135,136]. Stationary NESS probability currents from knot types *K* to $K^{\prime }$ were then calculated using Equations (4) and (6),

(8) $$i^{*}\left(K\to K^{\prime }\right)\equiv \frac{1}{c_{0}}I^{*}\left(K\to K^{\prime }\right)=P^{*}\left(K\right)k\left(K,K^{\prime }\right)-P^{*}\left(K^{\prime }\right)k\left(K^{\prime },K\right)\;.$$

**Figure 7:**
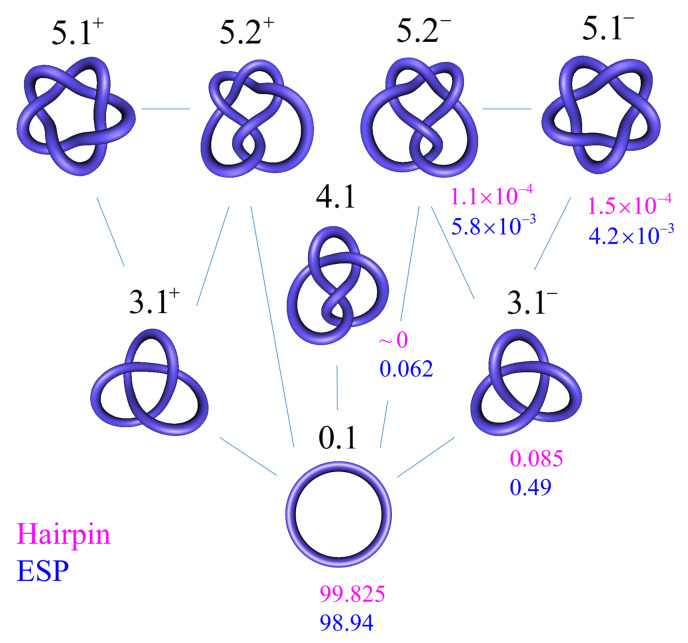
The fractions of knots with up to five crossings for 6-kbp torsionally unconstrained DNAs in the presence of type-II enzymes, and obtained by equilibrium strand passage (ESP). The numbers that are shown for each knot *K* correspond to steady-state fractions $P^{*}\left(K\right)$, in percent, for DNAs that undergo type-II enzyme activity with a hairpin-like G segment (magenta) along with the equilibrium fractions $P_{eq}\left(K\right)$ obtained by ESP (blue). The fractions for the right-handed isoform of a chiral knot are the same as for the left-handed isoform by symmetry. For type-II enzymes, only transitions between the knots connected by straight lines occurred, in accordance with one-passage connectivity (blue lines).

In Figure 7, we show steady-state fractions $P^{*}\left(K\right)$ for 6-kbp torsionally unconstrained DNA knots *K* with up to five crossings being obtained from our simulation results. This system is undergoing type-II-enzyme-catalyzed strand-passage reactions with a hairpin-like topoisomerase-bound G segment (cf. Figure 6). Knots with more than five crossings only occurred at a negligibly low frequency in our simulation and they were not considered. For type-II enzymes, only transitions between knots consistent with one-passage connectivity occurred [108,137]. In comparison, we also calculated the probabilities $P_{eq}\left(K\right)$ at thermal equilibrium. The latter were obtained from equilibrium-segment passage (ESP) ensembles, which were obtained in simulations that allow segments of circular chains (without a hairpin-like G segment) to freely pass through one another [103,132]. In agreement with reference [98], we found that the steady-state fraction of any knot different from the unknot is significantly reduced for type-II enzymes with hairpin-like G segment as compared to equilibrium (ESP) ensembles. For example, for the left-handed trefoil $3.1^{-}$, we found $P^{*}\left(3.1^{-}\right)/P^{*}\left(0.1\right)=8.5\times 10^{-4}$ (hairpin G segment) and $P_{eq}\left(3.1^{-}\right)/P_{eq}\left(0.1\right)=$0.0049 (ESP ensemble) (Figure 7), corresponding to a reduction by a factor of approximately 6 (compare Table 1 in reference [98], where both of the isoforms $3.1^{-}$ and $3.1^{+}$ were included in the statistics of the trefoil knot 3.1 for 7-kbp DNAs). The difference in reduction factors of 6 in our study and 14 in reference [98] can be explained by the fact that the hairpin-like G segment that was considered in reference [98] had a 180 ${}^{\circ }$-bend across the four segments comprising the G segment, when compared to a smaller 120 ${}^{\circ }$-bend in our model (Figure 6).

### 3.2. Knot-Resolution Pathways in Torsionally Unconstrained DNA

In the absence of a biological process that actively delivers a complex knot type to the ensemble of circular DNA molecules, type-II enzyme action will result in steady-state probabilities $P^{*}\left(K\right)\ll P_{eq}\left(K\right)$ for any knot *K* different from the unknot (Figure 7), as discussed in Section 3.1. Thus, practically no knotted DNAs appear for DNA molecules a few kbp in length. However, a typical situation in vivo is that some biological process is present that actively generates knotted DNAs, and type-II enzymes are essential in removing these knots (cf. Section 1.4 and Figure 2 and Figure 3).

In order to address this biologically relevant situation, we now assume the presence of an extrinsic process that continuously delivers DNA molecules to the system in a complex knotted state, which we designate as a *source knot*, $K_{S}$. In this case, the resulting steady-state probabilities $P^{*}\left(K\right)$ are appreciable for the source knot $K_{S}$ and all intermediate knot types *K* along the unknotting pathway that is generated by topoisomerase-II action. To illustrate this effect, we consider a 6-kbp circular, torsionally unconstrained DNA (due to nicks or type-I enzyme action) acted on by a type-II enzyme at a hairpin-like G segment. The type-II enzyme activity takes place concomitantly with a process that continuously converts unknotted DNA to DNA that form the source knot $K_{S}=10.139^{-}$ at a constant rate $k_{S}$. The DNA molecules that are delivered by the external process as the source knot are converted via strand passages induced by type-II enzyme action to simpler knots in a stepwise manner, which results in a pathway of intermediate knots. Each round of type-II enzyme action either leaves the knot type *K* of the DNA substrate unchanged, or converts *K* to a different knot type $K^{\prime }$ that is consistent with one-passage connectivity [108,137]. The DNA molecules are eventually converted back to the unknot, which results in a cyclic process of knot conversions that is driven by the external biological process and type-II enzyme action. The cycle process is characterized by non-equilibrium steady-state (NESS) probabilities $P^{*}\left(K\right)$ of DNA molecules having knot type *K*, and NESS transition currents $i^{*}\left(K\to K^{\prime }\right)$ from *K* to $K^{\prime }$ (Equation (8)).

**Figure 8:**
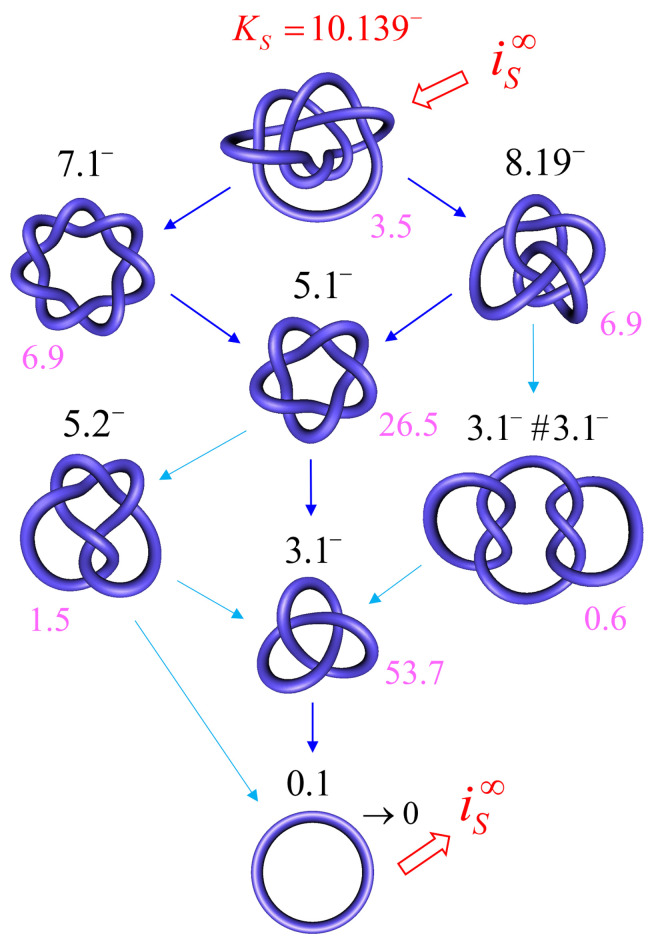
Unknotting pathways for 6-kbp circular, torsionally unconstrained DNA acted on by a type-II enzyme with hairpin-like G segment, in the presence of a process that converts unknotted DNA to DNA forming a source knot $K_{S}=10.139^{-}$ at a constant rate $k_{S}$. The pathways are shown for the limit of large source rate $k_{S}$ for which the steady-state probabilities and currents approach finite values $P^{*}\left(K\right)$ and $i^{*}\left(K\to K^{\prime }\right)$, respectively. The dominant currents with $i^{*}\left(K\to K^{\prime }\right)/i_{S}^{\infty }>0.1$ are shown as dark blue arrows and subdominant currents with $0.05<i^{*}\left(K\to K^{\prime }\right)/i_{S}^{\infty }<0.1$ are shown as light blue arrows. Steady-state probabilities $P^{*}\left(K\right)$, in percent, are shown next to each knot type *K* (magenta).

Figure 8 shows the resulting unknotting pathways in terms of intermediate knot types *K* and transition currents $i^{*}\left(K\to K^{\prime }\right)$ (blue arrows). The biological process that generates the source knot $K_{S}=10.139^{-}$ is modeled by a constant, externally imposed source rate $k_{S}=k\left(\mathrm{unknot},K_{S}\right)$ in Equation (1), which results in a constant source current $i_{S}$ from the unknot to $K_{S}$. We found that, for large source rates $k_{S}$, the resulting source current converges to a constant value $i_{S}^{\infty }$; in Figure 8, we use this finite limit source current $i_{S}^{\infty }$ and include all knots *K* for which the steady-state current $i^{*}$ passing through *K* is at least 5% of the source current $i_{S}^{\infty }$. The steady-state probabilities $P^{*}\left(K\right)$ are shown, in percent, next to each knot type *K* for type-II enzymes with the hairpin G segment. Interestingly, only a very small number of intermediate knots *K* contribute significantly to the unknotting pathway, even though there are about 250 different knot types with 10 or fewer crossings [103]. Note that, in the limit of large source rate $k_{S}$, the unknot 0.1 is depleted by the biological process, which implies that the steady-state probability of the unknot vanishes as $P^{*}\left(0.1\right)∼1/k_{S}$. Conversely, for all other knot types, the steady-state probabilities and currents approach finite values $P^{*}\left(K\right)$ and $i^{*}\left(K\to K^{\prime }\right)$ in the limit of a large source rate $k_{S}$, respectively.

### 3.3. Knot-Resolution Pathways in Knotted, Supercoiled DNA

We now turn to the case of covalently closed, circular duplex DNA for which the degree of supercoiling is described by a given value of the linking number difference relative to relaxed DNA, $\Delta Lk=Lk-Lk_{0}$. Because ΔLk is conserved in circular DNA in the absence of a process that cuts one of the two strands of the DNA double helix, ΔLk is a topological property of the DNA molecule. Thus, two parameters describe the topological state of covalently closed DNA circles, namely the knot type *K* and the linking number difference ΔLk (Section 1). In what follows, these topological states will be denoted as a=(K,ΔLk).

For DNAs in the size range considered here and in the absence of a process that actively delivers a complex knot type, the equilibrium probabilities $P_{eq}\left(K,\Delta Lk\right)$ are very small for any knot *K* different from the unknot. Thus, practically no knotted DNAs appear for single, uncatenated DNA molecules at thermal equilibrium. In the presence of type-II enzymes and ATP, these probabilities are reduced even further. However, the presence of an extrinsic process that continuously delivers DNA molecules in a complex source state $a_{S}=\left(K_{S},\Delta Lk_{S}\right)$ results in steady-state probabilities $P^{*}\left(a\right)$ that are appreciable for the source state $a_{S}$ and all the intermediate states a=(K,ΔLk) along the topoisomerase-dependent knot-resolution pathway, as discussed in Section 3.2 for the case or torsionally unconstrained DNA. To illustrate, we assume that a process is present in the ensemble of 6-kbp duplex DNAs that continuously converts unknotted DNAs with ΔLk=0 to the source knot $K_{S}=10.139^{-}$ with linking number $\Delta Lk_{S}=-12$ and at a constant rate $k_{S}$.

The DNA molecules that are delivered in the source state $a_{S}=\left(10.139^{-},-12\right)$ by the extrinsic process are converted by type-II enzyme strand passages to simpler topological forms in a stepwise manner, resulting in a pathway of intermediate topological states. Each round of type-II enzyme action converts a DNA substrate in the state (K,ΔLk) to a product state $\left(K^{\prime },\Delta Lk^{\prime }\right)$ where $\Delta Lk^{\prime }=\Delta Lk\pm 2$ and $K^{\prime }$ is a knot that can be obtained from *K* by single passages of the DNA double helix (one-passage connectivity) [108,137]. Eventually the DNAs are converted back to the originating state (0.1,0), i.e., the unknot with ΔLk=0. The latter is then converted again to molecules in the source state $a_{S}=\left(10.139^{-},-12\right)$ by the extrinsic process, resulting in a continuous cycle. The cyclic process is characterized by non-equilibrium steady state (NESS) probabilities $P^{*}\left(K,\Delta Lk\right)$ for DNAs in topological states a=(K,ΔLk), and NESS currents $i^{*}\left(a\to b\right)$ for transitions from states a=(K,ΔLk) to $b=(K^{\prime },\Delta Lk^{\prime })$.

**Figure 9:**
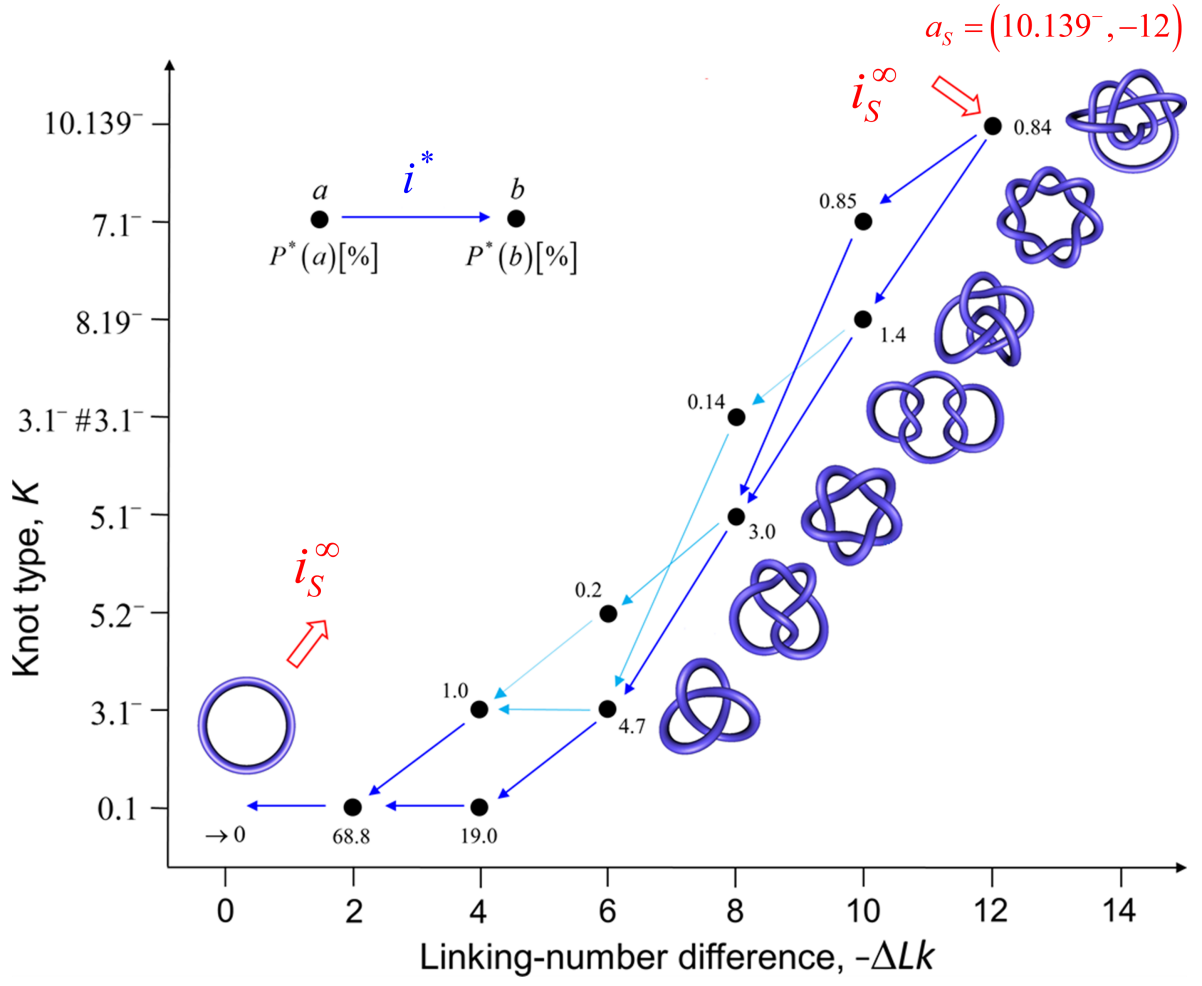
Unknotting pathway (black dots / numbers) and probability currents (blue arrows) that are generated by type-II enzymes modeled as a hairpin-like G segment in the space of topological states (K,ΔLk). We imposed an external process that converts unknotted DNA with ΔLk=0 to DNA forming a source knot $10.139^{-}$ with ΔLk=−12 in the limit of a large source rate $k_{S}$. The dominant and subdominant currents are indicated by dark blue and light blue arrows, respectively (cf. Figure 8).

Figure 9 shows the resulting unknotting pathway for type-II enzymes modeled by a hairpin-like G segment. Steady-state probabilities $P^{*}\left(a\right)$, in percent, are shown next to each state a=(K,ΔLk) (filled circles), and NESS currents $i^{*}\left(a\to b\right)$ are indicated by dark blue and light blue arrows (cf. Figure 8). The steady-state probabilities $P^{*}\left(a\right)$ were calculated using a chemical master equation, as described in Section 2.1, where, now, the transitions are between topological states a=(K,ΔLk). To this end, ΔLk was kept fixed during the simulation of the semiflexible, discrete wormlike chain model for duplex DNA, and the mechanical potential energy due to the torsional strain was calculated from the deviation of the twist from mechanically relaxed DNA, ΔTw. The latter was calculated for each chain conformation by using White’s equation ΔTw=ΔLk−Wr, where Wr is the writhe of the chain conformation [68,130,131,132]. The results shown in Figure 9 suggest that the canonical type-II mechanism is more efficient in unknotting than supercoil relaxation, a conclusion that could not have been obtained while using previous computational approaches.

## 4. Discussion

The mechanism and thermodynamics of DNA topology simplification is critical in understanding how DNA topology is regulated in a broader biological context (Section 1.4). Our model for the dynamics of type-II enzyme driven transitions in DNAs that are both knotted and supercoiled complements previous studies conducted on nicked, knotted DNAs, and unknotted, supercoiled DNAs, respectively [5,53,68,98,99,100,101,102,103]. Thus, our approach comprehensively and simultaneously addresses the kinetics of superhelix relaxation and knot resolution. For illustration, we considered knot resolution pathways in knotted, supercoiled DNA, in which an extrinsic biological process continuously delivers DNA in a complex source state with knot type $K=10.139^{-}$ and linking number ΔLk=−12 (Figure 9). For comparison, we also studied knot resolution pathways in knotted, nicked DNA (Figure 7 and Figure 8). Our approach that is based on a chemical master equation, such as Equation (1), is completely general, and it describes not only steady states, but also the time-dependent dynamics of transitions between topological states in DNA that are catalyzed by type-II enzymes and other enzymes, such as site-specific recombinases (Section 1).

A novel feature of our model is its capability to dynamically account for processes that generate complex knots in vitro or in vivo. This is significant because topoisomerases maintain the integrity of genomic DNA during transcription and replication, requiring the relaxation of (+) and (−) supercoils that respectively build up ahead of and behind RNA- and DNA-polymerase complexes (Figure 3). At the same time, DNA can become knotted via type-II-dependent and independent pathways through recombination and other processes in the cell (Section 1.4). Our analysis complements recent work conducted by Shimokawa et al., who considered stepwise unlinking of DNA-replication catenanes by the Xer site-specific recombinase [79]. Indeed, our approach can be generalized to quantitatively analyze the rates of circular-DNA linking and unlinking.

Our model reproduces the experimental observation that type-II topoisomerases remove crossings in trefoil DNA knots that are below the level expected at thermal equilibrium [53] (Figure 7). For knotted, supercoiled DNA, our results unexpectedly show that the type-II mechanism is generally and substantially more efficient at unknotting than supercoil relaxation. This is because, for many knots, the free-energy gradient is larger in the direction of knot simplification than in the orthogonal direction of superhelix relaxation (cf. Figure 4 of [103]). These advancements in our understanding of type-II mechanism would not have been possible without integrating the conformational statistics of knotted supercoiled DNA with the dynamics of defined topological states.

It has long been argued that type-II topoisomerases use the free energy of ATP hydrolysis to simplify DNA topology that is below the level at thermodynamic equilibrium [39,42,52,100,102,138]. An interesting question is how and at which point during the reaction cycle the enzyme achieves this (cf. Figure 4A,B). It should be noted that the enzyme is able bind to DNA and perform strand passage, even without ATP hydrolysis [113,139]. Early studies suggested that the hydrolysis of two ATP molecules occur during the reaction cycle, the first before intersegmental transport and the second at the end of the reaction [140,141]. According to this model, the energy of ATP hydrolysis of the second reaction is used to dissociate the stable DNA-enzyme complex after the reaction and reset the enzyme for the next reaction cycle. More recently, it was pointed out that additional free energy consumption occurs at several points during the reaction cycle [102,138]. For topoisomerase-IV mutants, it was found that free energy consumption is associated with the bending of the DNA G segment by the enzyme (Figure 4A,B) [40]. Further, it has been observed that the affinity of type-II enzymes is greater for supercoiled DNA than for relaxed or nicked DNA, possibly because the former is more bent, on average, than the latter, which reduces the net thermodynamic cost of forming a bent G segment during the binding of the enzyme [142]. We hope that our quantitative biophysical approach will shed light on the many open questions that are related to the regulation of DNA topology in the cell by stimulating the experimental work and theoretical/computational modeling of topoisomerases and other enzymes that alter DNA topology.
